# Stripping Voltammetry in Trace Ga(III) Analysis Using Different Working Electrodes: A Review

**DOI:** 10.3390/ma18040769

**Published:** 2025-02-10

**Authors:** Malgorzata Grabarczyk, Edyta Wlazlowska, Marzena Fialek

**Affiliations:** Department of Analytical Chemistry, Institution of Chemical Sciences Maria Curie−Sklodowska University, 20-031 Lublin, Poland; malgorzata.grabarczyk@mail.umcs.pl (M.G.); marzena.fialek@mail.umcs.pl (M.F.)

**Keywords:** gallium, stripping voltammetry method, working electrode, modified electrodes, complexing agent, interferences

## Abstract

Heavy metal contamination of water has become a global environmental problem in recent years, which is caused by the rapid development of economies and industries. Gallium is of enduring interest because of its wide range of applications in technology and industry. In its pure form or as a component of alloys, gallium is used in devices such as high-current switches, pressure gauges, and thermometers. Gallium compounds also play an important role in electronics and optoelectronics, particularly in devices that operate in the infrared range. Gallium isotopes are also used in medical diagnostics. The increasing demand for gallium emphasizes the need for accurate methods for its determination in different matrices. One method used for this purpose is stripping voltammetry. The working electrodes, complexing agents, and the influence of interferences on the accuracy of the measurement are discussed in detail, highlighting their crucial role in obtaining the analytical signal of gallium in procedures based on stripping voltammetry. Voltammetric procedures for the simultaneous determination of gallium and other metal ions are also described. The application of the developed procedures to the analysis of real samples is emphasized as crucial for environmental monitoring and the accurate determination of trace concentrations of gallium. A summary of the results is presented in the form of a table which provides detailed information on the stripping voltammetry methods, including the types of working electrodes, characteristics of the substrate electrolytes used, complexing agents, linear ranges, and detection limits. The table also includes accumulation times, interferences investigated, and practical applications of the methods discussed, making it a valuable resource for researchers and analysts involved in environmental analysis. The review highlights the importance of this technique as an accurate and sensitive tool for the analysis of gallium in environmental samples.

## 1. Introduction

In recent years, chemical pollution of waters and soils has increased due to the increased extraction and processing of mineral resources, the burning of energy resources, and the increased use of chemical fertilizers in agriculture. The main sources of trace element pollution are emissions from non-ferrous metal plants, coal-fired power stations, and oil refineries. The cause of this phenomenon is the low efficiency of dust collection systems and the use of inappropriate technology. Elements released during technological processes enter the environmental cycle and the environment. High levels of these elements in the environment affect the functioning of plant and animal ecosystems [1,2,3].

Gallium is a relatively rare element in the Earth’s crust. Although gallium is not abundant in the Earth’s crust, it can be found in various minerals such as bauxite, kaolinite, and zinc ores. It is usually obtained as a by-product from the processing of aluminum and zinc. It is a fascinating and relatively rare metal that has unique properties and a variety of applications in different fields [4,5]. Discovered in the 19th century, gallium has found numerous technological and industrial applications. Gallium is a silvery-white metal at room temperature, and its melting point is just above 29.7 °C. This low melting point means that gallium literally melts in your hand when exposed to body heat. It is a relatively reactive element, capable of forming compounds with other elements, similar to aluminum and indium. It does not corrode easily, making it useful in some specialist applications. Commonly used gallium compounds include gallium arsenide and gallium nitride. They are used as semiconductor materials in electrical devices such as high-frequency integrated circuits, laser diodes, LEDs, and solar cells. Its electrical properties make it suitable for electronic and optoelectronic applications. In addition, gallium finds application in medical diagnostics, where its isotopes are used as contrast agents in diagnostic imaging. Radioactive gallium and stable gallium nitrate are also used clinically as diagnostic and therapeutic agents in the treatment of cancer [5,6,7,8,9,10].

Irrespective of their use in medicine or electronics, gallium compounds are characterized by a certain degree of toxicity. Patients undergoing gallium nitrate therapy for a variety of conditions may benefit from this treatment. Although these therapies can be effective, there is a risk of clinical toxicity if doses are not precisely controlled. The therapeutic index, i.e., the ratio of a toxic dose to a therapeutic dose, must be carefully determined to minimize the risk of adverse effects such as kidney or liver damage. Animal studies have shown that exposure to gallium arsenide can cause toxic effects in several organ systems, including the respiratory, liver, and nervous systems. For this reason, people working in environments where gallium arsenide is used must be protected from the potential risk of poisoning by wearing appropriate personal protective equipment and by monitoring the levels of this compound in the workplace. In conclusion, although gallium and its compounds have a wide range of applications, their toxicity requires special attention. In both medicine and industry, it is essential to ensure that appropriate safety measures are in place and that potential risks are monitored to minimize the health risks associated with exposure to these substances. Therefore, accurate monitoring of its concentrations in different matrices such as water, soil, or biological tissues is essential [11,12,13,14,15].

The impact of trace elements on the environment, and therefore on human health, has encouraged the development of analytical techniques and instruments capable of measuring low concentrations. A number of analytical techniques have been described in the literature for the determination of traces of gallium in environmental samples, biological samples, or synthetic samples. Methods commonly used for the determination of gallium include spectrophotometry [16,17], spectrofluorimetric [18,19,20], atomic emission spectroscopy (AES) [21], graphite furnace atomic absorption spectroscopy (GFAAS) [22,23,24], flame atomic absorption spectrometry (FAAS) [25], inductively coupled plasma mass spectrometry (ICP−MS) [26,27,28,29,30], and inductively coupled plasma emission spectroscopy (ICP−OES) [31,32]. Most of these methods are expensive, requiring specialized equipment, significant amounts of reagents, highly skilled operators, and complex and time-consuming sample preparation. Therefore, techniques that can be performed on inexpensive equipment, have low reagent consumption, and require small sample volumes are desirable. Stripping voltammetry is an example of such a method. The simplicity, high selectivity, and sensitivity of these methods make them readily used for the determination of trace amounts of substances. In stripping voltammetry, measurements are carried out in two stages, the first being pre-concentration, in which the analyte is collected on the working electrode, and stripping, in which the collected analyte undergoes a reaction as a result of a change in electrode potential, accompanied by the recording of a voltammogram. In anodic stripping voltammetry (ASV), the electrolysis process is carried out by electrochemical reduction of the analyte at the electrode surface. The analyte, which is in ionic form in solution, is reduced and pre-concentrated at the electrode surface for subsequent analysis. In adsorptive stripping voltammetry (AdSV), analyte pre-concentration occurs by the adsorption of complexes of the labeled ion with a suitable complexing agent on the electrode surface. This process involves the formation of stable analyte complexes that adsorb onto the electrode surface, increasing the sensitivity and selectivity of the method. Cathodic stripping voltammetry (CSV) is a third method in which the accumulation of analyte takes place through the formation of a difficult, soluble compound on the electrode surface. This process enables the detection of analytes that form insoluble compounds that are deposited on the electrode, allowing their accurate determination. [33,34,35,36,37,38]. Two stripping voltammetric techniques were used for the determination of gallium: ASV [39,40,41,42,43,44,45] and AdSV [46,47,48,49,50,51,52,53,54].

The present review of the literature on the determination of gallium by electrochemical methods covers several key aspects. The literature review covers all available publications on the determination of gallium (Ga(III)) by stripping voltammetry methods. For the adsorption stripping voltammetry (AdSV) method, the oldest article dates to 1986, and the most recent is from 2024. For anodic stripping voltammetry (ASV), publications from 1992 to 2020 were included. The analysis included both review and research articles identified in reputable databases. The review focuses on the discussion of supporting electrolytes and organic reagents used in voltammetric methods for the determination of gallium. In addition, the review includes an analysis of the use of electrodes in the determination of this metal. A discussion of one paper from the literature on the coulometric method for the determination of gallium is also included [55]. This paper reviews the methods for the stripping voltammetric determination of gallium described in the literature. In order to collect the most important information on these methods, Table 1 is presented, which contains key data on gallium determination procedures. It includes information on the methods used, the working electrodes, the detection limits obtained, the linearity ranges, and the accumulation time. Interferences affecting the gallium signal and the applicability of the developed methods to the analysis of real samples are also discussed.

Due to their wide range of negative potentials, high signal repeatability, excellent adsorption properties, excellent polarizability, and smooth surface, mercury electrodes were widely used until about 2000. However, with increasing awareness of the toxic properties of mercury, the use of mercury in the processes being developed has begun to be phased out. The determination of gallium by anodic stripping voltammetry (ASV) and adsorptive stripping voltammetry (AdSV) was performed using mercury-based electrodes such as a static mercury drop electrode (SMDE) [39,46], hanging mercury drop electrode (HMDE) [40,47,48], mercury film electrode (MFE) [42], and renewable mercury film silver-based electrode (Hg(Ag)FE) [43,49,50]. The most commonly used electrode in voltammetric measurements for the determination of gallium and other elements is the HMDE electrode. One of its advantages is the ability to precisely define the parameters of a mercury droplet, which directly affects the repeatability of its surface and, therefore, the repeatability of measurements. Among the disadvantages of these electrodes, in addition to their toxicity, is the need to use high-purity mercury to avoid contaminated mercury blocking the capillary. Mercury electrodes allow the lowest detection limits and have high reproducibility and repeatability of measurements, which is also noticeable when determining gallium with them.

The first procedures for the determination of gallium were carried out using mercury electrodes, such as SMDE and HMDE, for both ASV and AdSV methods [39,40,46,47,48]. The lowest detection limit to date was obtained using HMDE as the working electrode in the ASV method developed by Udisti et al. Although this method was developed some 36 years ago, other researchers have still not been able to lower the detection limit below that obtained in this study [40]. Table 1 compares the detection limits obtained with the different electrodes used in voltammetric measurements. The comparison also provides information on the methods used in each study, allowing an assessment of the effectiveness of different electrode types in achieving detection limits. The conclusion is that the choice of working electrode has a significant effect on the detection limits obtained. In the case of the mercury electrode, it can be seen that replacing it with less toxic electrodes results in higher detection limits.

Medvecký and Briančin were the first to use an electrode other than HMDE and SMDE for the determination of gallium(III) and indium(III) [41]. They suggested the use of a rotating glassy carbon electrode (RDE), which is used in the ASV method. In their research, they investigated the possibility of modifying the surface of this electrode by depositing films of mercury, bismuth, and a mixture of mercury and bismuth. With the formation of a mercury film on the RDE, the signal for Ga(III) is significantly reduced at higher concentrations, negatively affecting the accuracy and repeatability of the measurements. Therefore, modification with mercury is less effective and not recommended due to peak distortion at higher Ga(III) concentrations. The use of bismuth ions for modification has a positive effect on the signal obtained. The formation of a bismuth film allows the simultaneous determination of In(III) and Ga(III), and the determination of In(III) is possible over a wide concentration range with a linear dependence on peak height. The mixture of mercury and bismuth ions accumulated on the electrode surface can be successfully used for the determination of Ga(III) and In(III). Despite the presence of mercury, even at high Ga(III) concentrations, the signal is well-shaped, and its value is not reduced, as was the case with the first electrode modification. The best modifications for the simultaneous determination of gallium (Ga) and indium (In) on an RDE electrode are modifications using bismuth films and a mixture of mercury and bismuth. These modifications not only ensure high accuracy and repeatability of measurements but also minimize the toxicity of the materials used, an important step towards more sustainable laboratory practices. It is also worth noting that electrode modification with a bismuth film is particularly useful for routine environmental analysis where minimizing toxicity is critical. On the other hand, a mixture of mercury and bismuth may be more suitable in situations with the highest sensitivity and accuracy. These modifications allow analytical procedures to be tailored to specific research needs and requirements [41].

In the study of [42], a glassy carbon electrode was again used as the electrode, which was additionally modified during the measurement. However, the study showed that in order to obtain a signal from Ga(III), it was necessary to modify the surface of the GCE electrode with mercury. Without the presence of mercury ions in the solution, the signal did not appear on the voltammogram, indicating that the electrode needed to be modified for Ga(III) detection. The addition of Hg(II) to the test solution resulted in the formation of a mercury film on the surface of the GCE electrode. When the mercury ion concentration in the test solution was 1 × 10^−3^ g L^−1^, an adequate film thickness was achieved. An addition of this amount of mercury may be considered low in the context of some laboratory applications, but even at this level, mercury is toxic and potentially harmful to the environment and human health [42].

Another electrode used for gallium determinations to reduce the amount of mercury used in the study was the Hg(Ag)FE electrode. Three publications [43,49,50] have described and used this electrode for the determination of gallium. In [49], the signal obtained on the Hg(Ag)FE electrode was compared to that on the HMDE electrode, showing a 16-fold increase in gallium signal for the Hg(Ag)FE electrode compared to the HMDE. Despite the higher background current for the Hg(Ag)FE electrode, the signal-to-background current ratio is more favorable, which is a significant advantage for this electrode. Another advantage of the Hg(Ag)FE electrode is that the working area can be easily adjusted over a wide range, allowing it to be adapted to different measurement conditions [49]. The mercury layer is regenerated by moving the base of the silver electrode inside the electrode body, ensuring that the silver wire is in contact with the liquid amalgam twice during each cycle. All the amalgam is replaced after approximately 2000 cycles, and the regeneration time is less than one second. The Hg(Ag)FE electrode has high mechanical durability and measurement repeatability, making it a practical and reliable tool for gallium electrochemical analysis [43,49,50].

The solid bismuth microelectrode (BiFµE) has been developed as a safe and versatile alternative to mercury electrodes, enabling measurements over a wide potential range while eliminating the risk of mercury toxicity [45]. In an optimized procedure for the determination of Ga(III), the BiFµE electrode successfully achieved a detection limit of 7.0 × 10^−9^ mol L^−1^. The study showed that the electrode had to be activated by adding bismuth ions to the solution, which were reduced at corresponding potentials applied to the electrode. Measurements without this step resulted in a significant drop in signal, prompting the authors to include activation as a key element of the measurement procedure [45].

Another electrode for the determination of gallium, in which bismuth was the basis of the working electrode, was a film bismuth electrode (BiFE) [44]. The formation of a bismuth film on the GCE surface was carried out using an in situ method directly from the test solution. A key aspect of the process was to ensure that the reaction environment was not conducive to the hydrolysis of bismuth ions, which could hinder film formation on the electrode surface. For this reason, a pH 4.6 acetate buffer was used as the supporting electrolyte, providing the right conditions for efficient bismuth deposition. In an acidic or slightly acidic environment, bismuth ions remain stable in solution, which promotes their deposition on the electrode surface in the form of a homogeneous metallic film. Under these conditions, the electrochemical process leading to the reduction of bismuth ions to metal occurs efficiently, without interference from competing reactions. In contrast, in neutral or alkaline environments, bismuth ions hydrolyze to form bismuth hydroxides or other derivatives that are difficult to dissolve. These insoluble precipitates are deposited in solution or on the electrode, preventing the formation of a uniform metallic film. Both the concentration of bismuth ions required to form the film and the analytical parameters under which the process took place were optimized. Kamat et al. also carried out a comparative study using BiFE and MFE electrodes to assess the differences in the signals generated by gallium(III). Measurements made under identical conditions and at the same gallium concentrations showed that the signals were similar in height but different in shape—the peak on the BiFE electrode was broader and slightly shifted toward more negative potentials. The calibration curve confirmed that the BiFE electrode had better linearity and a lower detection limit of 9.5 × 10^−^⁸ mol L^−1^, while the detection limit for the MFE electrode was 1.1 × 10^−^⁷ mol L^−1^. Therefore, the BiFE electrode was recommended as the better option of the two electrodes compared [44].

Another procedure also used a glassy carbon electrode (GCE) modified by forming a metallic film on its surface, which, in this case, is a lead film [52]. This film was produced using the in situ method, which is an important advantage as it does not increase the time taken for a single measurement. One of the key advantages of this electrode is that the materials used to form the film are less toxic compared to mercury electrodes. The detection limit for Ga(III) determination with PbFE was 3.8 × 10^−9^ mol L^−1^, indicating the high sensitivity of this method compared to other electrodes, including mercury. PbFE electrodes, unlike BiFE, can be fabricated in a variety of environments, but an acidic environment is most commonly used. Measurements were carried out in a solution of 0.05 mol L^−1^ acetic acid, which was chosen as the optimal supporting electrolyte for the accumulation of the Ga(III)−cupferron complex on the lead film. The study showed that the gallium signal appeared only in the presence of acetic acid or its mixture with sodium acetate. The maximum peak current was observed at 0.05 mol L^−1^ CH_3_COOH. At higher pH, the gallium signal weakened, and the shape of the peaks deteriorated, they became broader, and their potential shifted to more negative values. In addition, an increase in acetic acid concentration above 0.05 mol L^−1^ caused a decrease in the gallium peak current. Therefore, acetic acid at a concentration of 0.05 mol L^−1^ was selected as the best-supporting electrolyte [52].

The latest method for the determination of gallium is based on the use of a working electrode consisting of multi-walled carbon nanotubes and spherical glassy carbon powder (MWCNT/SGCE) [54]. This environmentally friendly electrode has been further modified by the addition of a lead film. In the absence of electrode modification, no signal from gallium was observed due to the fact that the Ga(III)−cupferron complex was not adsorbed on the surface of the working electrode. Therefore, the presence of lead ions in solution was essential for the formation of a film that allowed the complex to accumulate, resulting in the appearance of a characteristic peak for gallium on the voltammogram. The choice of this electrode proved to be extremely successful as the detection limit obtained is the second lowest of any existing method for the determination of gallium. By using MWCNT/SGCE as the substrate instead of GCE, the limit of determination of Ga(III) was increased from 3.8 × 10^−9^ mol L^−1^ to 9.5 × 10^−10^ mol L^−1^. Of the methods used, only the method using the HMDE electrode achieved a lower detection limit than that obtained with the MWCNT/SGC electrode. This is a major advantage of the newly developed method, as it allows the accurate determination of gallium while maintaining the environmentally friendly nature of the electrode. An additional benefit is the elimination of toxic materials, making the process safer and more versatile in analytical applications [54].

This section discusses the use of electrodes in anodic stripping voltammetry (ASV) and adsorptive stripping voltammetry (AdSV) methods for the determination of gallium (Ga). Both classical mercury electrodes and modified electrodes, which play a key role in achieving high sensitivity and precision measurements, are presented. Mercury electrodes such as HMDE, SMDE, and MFE were commonly used in both methods, but due to the toxicity of mercury, their use has gradually decreased in favor of more environmentally friendly alternatives. Of particular importance in the ASV method were electrodes such as SMDE and HMDE, which enabled very low detection limits. In the AdSV method, modified electrodes such as MWCNT/SGCE offer greater reproducibility of results and maintain high sensitivity while reducing the risk of toxicity, making them particularly useful for environmental analysis. The use of different electrode solutions allows them to be tailored to the specific needs of both ASV and AdSV methods, which has allowed the development of a number of voltammetric methods for the determination of gallium(III). With these innovative approaches, it is possible to perform accurate analyses while maintaining safety and sustainability in analytical laboratories.

## 2. Role of Complexing Agent for Ga(III) AdSV Determination

Based on the existing literature review on the electrochemical determination of gallium, it can be concluded that the choice of a suitable complexing ligand is crucial for sensitive and selective determination by adsorptive stripping voltammetry (AdSV). These ligands not only increase the sensitivity of the method but also improve its selectivity in the presence of interfering ions. Therefore, further research into new ligands and the mechanisms of their interaction with gallium can contribute significantly to the development and optimization of these analytical techniques [55]. In the adsorptive stripping voltammetry used for the determination of gallium, various complexing agents were used, such as solochrome violet RS (SVRS) [46], ammonium dithiocarbamate (APDC), pyrocatechol violet (PCV), dithiocarbamate dithion (DDTC) [47], catechol [49,50], alizarin red S (ARS) [51] and cupferron [48,52,53,54]. Each of these complexing agents has specific properties that enable efficient complexation of gallium, which is essential for reliable analytical results.

In the adsorptive voltammetric determination of gallium, the complexing agent used for the first time was solochrome violet RS (SVRS) [46]. SVRS proved to be an effective ligand for Ga(III) complexation, allowing the complex to accumulate on the surface of the mercury droplet electrode. The Ga(III)−SVRS complex shows two characteristic reduction peaks, allowing the accurate determination of gallium at very low concentrations. With relatively short accumulation times, detection limits of 1.1 × 10^−9^ mol L^−1^ can be achieved for gallium. The experiments were performed in acetate buffer at pH 4.8. The chosen SVRS concentration was 2 × 10^−6^ mol L^−1^ since the maximum reduction signal of the complex was obtained at this concentration. Analysis of the effect of SVRS concentration on the magnitude of the reduction current showed that the intensity of the second reduction peak increased rapidly with increasing SVRS concentration to about 1.2 × 10^−6^ mol L^−1^, after which the signal stabilized. The accumulation potential of the Ga−SVRS complex also significantly affected the reduction signal obtained. At potentials in the range of −0.5 V to −0.9 V, too little adsorption of the complex was observed, resulting in a lower signal. On the other hand, at potentials more positive than −0.4 V, there was competition from the free dye for adsorption sites on the electrode surface, which also led to a weaker signal. The optimal accumulation potential was found to be −0.4 V, at which the highest reduction signal of the Ga−SVRS complex was obtained [46].

As shown in subsequent works, another complexing agent allowing effective and accurate determination of trace amounts of gallium is catechol [49,50]. In these procedures, a renewable mercury film silver-based electrode (Hg(Ag)FE) was used as the working electrode on which the accumulation occurred. The addition of catechol to the supporting electrolyte significantly increases the Ga(III) peak current, indicating the efficient formation of a Ga(III)−catechol complex. Catechol concentration has been shown to affect gallium peak potential. As the catechol concentration increases, the gallium peak potential shifts to more negative values. The optimal catechol concentration, in this case, was 3 × 10^−3^ mol L^−1^, which gave the best analytical results in terms of signal intensity and stability. The method was shown to have good precision and to be suitable for accurate chemical analysis of gallium [49]. In contrast, the use of catechol as a complexing agent was essential in reference [50], where the simultaneous determination of gallium and germanium by the AdSV method was carried out. Measurements carried out in the absence of catechol showed no signals on the recorded voltammograms. The need to select the optimum concentration of catechol was crucial, as higher concentrations of complexing agent caused a decrease in the signal from Ge(IV) and an increase in the peak from Ga(III), which could affect the accuracy of the assays. The use of a concentration-optimized catechol enabled the efficient simultaneous determination of traces of gallium and germanium by the AdSV method, providing high sensitivity, selectivity, and precision measurements [50].

Based on the work of Li et al., the use of alizarin red S (ARS) as a complexing agent for the determination of gallium can be analyzed [51]. ARS forms complexes with Ga(III) which can be adsorbed on the surface of a carbon paste electrode (CPE). The Ga(III)−ARS complex shows a reduction peak at a potential of −0.52 V, corresponding to an irreversible reduction of the ARS ligand bound in the complex. The optimal environment for the analysis was a buffer of pH 4.5, as the maximum peak current was obtained in this range. The study showed that for pHs above 4.3, the peak current increased with increasing pH, and above 4.8, it decreased significantly, which could be indicative of Ga(III) hydrolysis. Analysis of the effect of ARS concentration on the peak current showed that the peak current increased with increasing ARS concentration up to a value of 8.0 × 10^−6^ mol L^−1^ and then decreased at higher concentrations. The decrease in signal could be attributed to competition between free ARS and the Ga(III) ARS complex for adsorption sites on the electrode surface. The detection limit of gallium in this case was 1.4 × 10^−10^ mol L^−1^ with an accumulation time of 180 s and an ARS concentration of 1.0 × 10^−5^ mol L^−1^. The value obtained demonstrates the high sensitivity of the developed method [51].

In reference [47], three complexing agents, namely ammonium dithiocarbamate (APDC), pyrocatechol violet (PCV), and dithiocarbamate dithion (DDTC), were tested for their suitability for gallium determination using a hanging mercury drop electrode (HMDE) as the working electrode. The focus was on optimizing the experimental conditions and analyzing the three complexing agents in order to select one that provided a lower detection limit for gallium. The study showed that the Ga(III)−DDTC complex provides the lowest detection limit of 1.3 × 10^−9^ mol L^−1^, making DDTC the most effective of the three complexing agents tested. The optimal environment for this complex was acetyl acetate buffer pH 4.5 with a DDTC concentration of 1.0 × 10^−5^ mol L^−1^, which gave the best reduction signal with minimal background. For the Ga(III)−PCV complex, the Britton−Robinson buffer was found to be the most favorable environment, and the optimum concentration of PCV was 8.0 × 10^−6^ mol L^−1^. The detection limit for gallium with this complexing agent was 9.9 × 10^−9^ mol L^−1^. In contrast, for the Ga(III)−APDC complex, acetate buffer at pH 4.5 proved to be the best environment, and the APDC concentration was 1.0 × 10^−5^ mol L^−1^, giving a detection limit of 5.0 × 10^−8^ mol L^−1^. Of the complexing agents tested, DDTC showed the lowest detection limit and the best analytical properties, making it the preferred choice for the determination of traces of gallium [47].

Based on the available data, cupferron was the most frequently chosen complexing agent for the determination of gallium by adsorptive stripping voltammetry, being used in no less than four papers [48,52,53,54]. In these studies, different working electrodes were used: the paper [48] used a hanging drop mercury electrode (HMDE), the paper [52] used a lead film electrode (PbFE), the paper [53] used a bismuth film electrode (BiFE), and the paper [54] used a multiwall carbon nanotubes/spherical glassy carbon electrode (MWCNT/SGCE). Cupferron is the most commonly chosen complexing agent for the determination of gallium by the AdSV method due to its efficiency, high sensitivity, and selectivity. Precise and accurate determination of gallium in a variety of matrices is possible using stable Ga(III)−cupferron complexes and optimal experimental conditions. As a result, the cupferron is widely used in gallium determination studies, highlighting its importance in analytical methods. In reference [48], a cupferron concentration of 2.0 × 10^−4^ mol L^−1^ was used, and the experiments were carried out in the presence of acetate buffer at pH 3.0. It was observed that the signal decreased at higher pH of the solution, suggesting that changes in pH may affect the efficiency of complexation of gallium by cupferron and the intensity of the signal obtained during the measurements. Subsequently, references [52,53,54] used a cupferron concentration of 2.0 × 10^−4^ mol L^−1^, but the experimental environment varied from study to study. In [52], 0.05 mol L^−1^ acetic acid was used, whereas in [53,54] acetate buffer was used at pH 5.0 and pH 5.6, respectively. In addition, the lowest detection limit of the AdSV method was achieved with its use. When the MWCNT/SGCE was used as the working electrode, and the cupferron was used, the detection limit was 9.5 × 10^−10^ mol L^−1^ [54]. All these studies confirm that cupferron, thanks to its efficiency and flexibility in different experimental conditions, is an irreplaceable complexing agent that allows extremely accurate results in the determination of gallium by the AdSV method.

## 3. Interferences

In analytical methods used to determine ions in real samples, there is a risk of signal interference. The methods described in the literature for the determination of gallium by stripping voltammetry have taken into account the effects of foreign ions and organic matter on the gallium signal. However, these studies were not performed for all procedures. The effect of foreign ions has been investigated in papers [40,43,44,45,46,47,48,49,50,51,52,54], while the effect of organic substances has only been investigated in papers [45,46,48,49,50]. Studies have shown that the presence of other foreign ions and organic compounds in solution can significantly affect the accuracy and precision of measurements.

### 3.1. Influence of Metallic Interfering Ions

In references [40,43,44,45], in which Ga(III) determination procedures are performed using the ASV method, the interference of Cd(II), Cu(II), and Zn(II) ions was investigated. The addition of 30-fold excess Zn(II) did not cause signal perturbation in the study by Roberto Udisti and Giovanni Piccardi. The authors of this paper also demonstrated that a 1000-fold excess of Cd(II) and Pb(II) does not affect the Ga(III) signal. Only a 100-fold excess of copper ions interfered with the signal, lowering its value. The reason for this interference is the formation of gallium−copper intermetallic compounds, resulting in the inability to determine gallium in the presence of copper [40]. The addition of a 1-fold excess of copper ions caused a 30% decrease in gallium peak height as measured by the method described in the paper [44]. A film bismuth electrode was used in this procedure, while a hanging drop mercury electrode was used in reference [40]. The film bismuth electrode has less toxic effects on the environment compared to the mercury electrode and better stability in the presence of some interfering ions, including copper(II). Therefore, the authors of this paper do not recommend the determination of gallium in the presence of Cu(II). An additional peak was recorded on the voltammetric curve when there was a 10-fold excess of cadmium(II) in the test solution. The cadmium peak did not interfere with the gallium signal, the peaks were well separated, and the height of the gallium peak was not changed. The disappearance of the signal on the voltammogram was noticed when a 2-fold excess concentration of zinc ions was added; only the signal of zinc was registered on the voltammogram [44].

On the other hand, gallium determination using a solid bismuth microelectrode in a 50-fold excess of copper(II) caused no signal interference. Using this electrode in the determination of gallium, only two elements caused interference with these ions, Fe(III) and Zn(II). Only a 2.5-fold excess of these ions caused no signal interference, while greater excesses of these ions relative to gallium caused interference [45]. The greatest decrease in gallium signal, up to 90%, was recorded using an electrode with Hg(Ag)FE in the presence of a 10-fold excess of Cu(II). With a 20-fold excess of Cu(II) in this procedure, the gallium signal disappeared completely. A 35% decrease in gallium signal was recorded when there was a 50-fold excess of zinc(II) ions in the solution. Piech et al. suggested eliminating Cu(II) and Zn(II) interference by adding sodium diethylenedithiocarbamate (DDTC) to the test solution to mask interfering ions in gallium determination. The ions interfering with the Ga(III) signal in this procedure appeared to be Fe(III) ions. The possibility of removing interference due to the presence of iron in the sample by adding F^−^ ions to the solution was also investigated. Removal of the iron interference by this method had a positive effect and made it possible to determine gallium in the presence of iron [43].

The papers [47,52] define the allowable concentrations of foreign ions that can be introduced into a solution containing gallium without interfering with the signal in adsorptive stripping voltammetry. Information on the ions tested as interferents in these methods can be found in Table 1, as it contains a column listing the interferents tested in each method. In studies using catechol as a complexing agent, vanadium(V), tellurium(IV), antimony(III), and iron(III) ions were found to interfere with the gallium signal, with a 1000-fold excess of Fe(III) causing the most interference, reducing the gallium signal by as much as 85% [49]. The selectivity of the CPE electrode was tested in the presence of ions commonly found in natural samples, added in 1000-fold, 200-fold, and 100-fold excess relative to the Ga(III) concentration. There was no significant decrease in the Ga(III) signal, except in the case of Fe(III) and Al(III), which caused interference and reduced the peak height of gallium [51]. The papers [49,51] proposed adding F^−^ ions to the test solution to eliminate interference. This method effectively neutralized the interference caused by Fe(III) and Al(III).

An evaluation of the effect of foreign ions on the signal of labeled gallium has also been performed in reference [48]. It was found that a 100-fold excess of bismuth(III), iron(III), mercury(II), and antimony(III) ions caused a 25% decrease in gallium signal. Much greater interference is caused by zinc(II), the same excess of which reduces the signal by up to 70%. The strongest effect was observed in the presence of a 5-fold excess of molybdenum(VI) and selenium(IV), which caused a 50% reduction in the gallium signal [48]. Gallium signal decay has also been observed with a 50-fold excess of molybdenum(VI) in a procedure using cupferron as a complexing agent [54]. Interference was also found in the presence of a 100-fold excess of Sb(III), Ti(IV), and V(V) ions, which caused a signal drop of 17%, 42%, and 57%, respectively [54].

In summary, the methods for the determination of Ga(III) by stripping voltammetry have shown different effects of foreign ions on the gallium signal. The most common perturbations were caused by Fe(III), Cu(II), Zn(II), and Al(III) ions, as well as Sb(III), Ti(IV), V(V), and Mo(VI). In particular, Fe(III) and Zn(II) caused a significant decrease in the gallium signal. Interference from copper ions was particularly severe in methods using the Hg(Ag)FE electrode. In contrast, ions such as Cd(II), Pb(II), and some metals in small excess such as Bi(III), Sb(III), and Te(IV) often did not significantly interfere with the signal, and their interference was easily neutralized. In many cases, the use of appropriate masking agents proved effective in eliminating interference from Fe(III), Cu(II), and other ions. The research highlights that electrode selection plays a key role in increasing the selectivity of the analytical procedure and reducing the influence of foreign ion interference, which is important for the accurate determination of Ga(III).

### 3.2. Influence of Organic Substances

Organic substances present in solutions can significantly interfere with or completely suppress the signal of the ion of interest in voltammetric measurements. This interference is due to the adsorption of these substances on the surface of the working electrode, blocking its active sites and making the initial accumulation of gallium impossible. References [43,45,46,48,49] discussed the effect of organic substances on the gallium signal, such as gelatin, humic acid (HA), fulvic acid (FA), and three types of surfactants: cationic—cetyltrimethylammonium bromide (CTAB), anionic—sodium dodecylsulfate (SDS), and nonionic—Triton X−100 surfactants. The presence of surface-active organic materials, such as gelatine, affects competitive adsorption on the mercury electrode surface, leading to interference with the gallium signal. The addition of gelatine to the gallium solution causes a decrease in the intensity of the first Ga/SVRS peak, while increasing its concentration causes the complete disappearance of the second peak, indicating strong interference from the adsorption of organic molecules [46].

In references [43,49], the effect of organic substances on the signal of the gallium to be determined was investigated using Hg(Ag)FE as the working electrode. Surfactant compounds such as Triton X−100 and humic acid have a significant effect on the results of voltammetric analyses, causing strong signal interference. The authors, therefore, chose these substances as interfering substances in their study. Using the anodic stripping voltammetry method, it was found that at a concentration of 0.1 mg L^−1^ Triton X−100, the gallium signal decreased by 14%, and higher concentrations resulted in a further decrease in the signal, reaching 97% at a concentration of 5 mg L^−1^ [43]. Studies based on the adsorptive stripping voltammetry method using catechol as a complexing agent showed similar results, but the signal dropped to 97% at a concentration of 1.5 mg L^−1^ [49]. Humic acid had a stronger effect on the gallium signal than Triton X−100 in both works. With the addition of 1 mg L^−1^ humic acid to the test solutions, the signal disappeared completely in the paper [43], while it decreased by 95% in the paper [49]. In both studies, it was observed that even low concentrations of these substances resulted in significant signal attenuation, while higher concentrations resulted in complete signal suppression. Therefore, prior to voltammetric analysis, these compounds should be thoroughly mineralized or destroyed, e.g., by digestion, to ensure the accuracy of the measurements [43,49].

When substances such as humic acid (HA), fulvic acid (FA), and natural organic matter (NOM) were added to the test solution using the BiFµE electrode, no signal drop was observed at concentrations up to 5 ppm, indicating that these substances do not affect the accuracy of gallium determination at such concentrations. Similarly, surfactants such as SDS and rhamnolipid did not affect the signal at the same concentration as the substances described above. In contrast, the presence of Triton X−100 and CTAB induced signal interference, confirming their potential to influence measurement results [45].

The paper [48] investigated the effect of the surfactants CTAB, SDS, and Triton X−100, as well as HA and FA, on the gallium signal using HMDE as the working electrode. The gallium signal was particularly affected by Triton X−100 and CTAB. Their presence in the solution caused significant signal interference, and in the case of Triton X−100 at 0.5 mg L^−1^ and CTAB, the gallium signal disappeared completely. No signal interference was observed with SDS, suggesting less interaction of these substances with the electrode surface. Grabarczyk and Wardak proposed eliminating the interference caused by these substances by introducing a sample mixing step with Amberlite XAD−7 and XAD−16 resin. Mixing the sample with the resin before voltammetric measurement allows unwanted organic compounds to be adsorbed onto the resin, effectively preventing them from interfering with the analysis result. The proposed procedure, which involves pre-mixing the sample with the resin prior to analysis, provides a quick and effective solution for eliminating interferences. This is an alternative to more time-consuming and complicated methods such as UV etching. This allows voltammetric analysis to be carried out more quickly, making it more practical, especially in analytical settings where time is of the essence. It also saves time and equipment, making it more accessible and efficient in daily analytical practice [48].

## 4. Simultaneous Determination of Ga(III) with Other Metal Ions

The simultaneous determination of heavy metals in environmental samples is crucial due to their widespread occurrence and potentially toxic effects on living organisms and ecosystems. Simultaneous monitoring of these metals is essential for assessing environmental quality and taking remedial action. Stripping voltammetry is a very effective analytical method that allows the simultaneous determination of ions at trace levels. This method combines electrochemical accumulation and analysis to achieve high sensitivity and selectivity. By using suitable working electrodes and complexing agents, it is possible to efficiently separate and determine individual metal ions in complex sample matrices [56].

An analysis of the literature shows that only a few methods have been described for the simultaneous determination of Ga(III) and other elements by strip voltammetry. Methods for the simultaneous determination of Ga(III) and In(III) [41,53], as well as Ga(III) and Ge(IV) [50], can be found in the literature. The first procedure described in the literature using stripping voltammetry methods with different types of thin-film electrodes (TFE) for the simultaneous determination of gallium with indium was proposed in a paper by Medvecki et al. [41]. The study used in situ generated thin-film electrodes on a glassy carbon electrode: a thin-film bismuth electrode (Bi TFE) and an electrode with a thin film of a mixture of mercury and bismuth (HgBi TFE). The simultaneous presence of both elements in the analyzed solution resulted in an interaction at the level of the anodic peaks. In order to minimize these interferences and obtain accurate results, the most appropriate method has been proposed, which is the method of standard additions at a constant concentration of the nonanalyzed element. Thanks to the high linearity of the dependence of the In peak height on the In(III) concentration, it was shown that the In(III) ion content could be accurately determined using a bismuth thin-film electrode (Bi TFE). The Ga(III) ion content, on the other hand, could be accurately determined using an electrode with a thin layer of a mixture of mercury and bismuth (HgBi TFE), which gave high linearity in the dependence of Ga peak height on Ga(III) concentration. The standard additive method, combined with the appropriate choice of electrode (Bi TFE for In(III) and HgBi TFE for Ga(III)), allowed accurate and reproducible determination of the concentrations of both elements in solution, minimizing mutual influence and interference [41].

Another method for the simultaneous determination of gallium and indium was described in [53]. In this study, bismuth ions were used to form a film on the surface of the working electrode. The bismuth film formation was carried out on a glassy carbon electrode during the accumulation of Ga(III)−cupferron and In(III)−cupferron complexes. The combination of the bismuth accumulation step and the labeled ions with a complexing agent allowed a single measurement to be performed rapidly, in just 30 s, greatly simplifying the procedure. With suitably selected concentrations of cupferron, bismuth ions, pH of the supporting electrolyte, and optimized analytical parameters, a clear separation of gallium and indium peaks was obtained in a single voltammogram. The indium peak appeared at a potential of approximately −0.745 V, while the gallium peak appeared at a potential of −1.025 V. Detection limits of 9.7 × 10^−9^ mol L^−1^ for gallium and 8.8 × 10^−9^ mol L^−1^ for indium were obtained [53].

The simultaneous determination of gallium(III) and germanium(IV) by adsorptive stripping voltammetry was proposed by Robert Piech [50]. A Hg(AgFE) electrode was used in this study. After optimizing the procedure, a voltammogram was recorded in which the Ga(III) and Ge(IV) peaks were separated. The Ga(III) peak appeared at a potential of −1.07 V, while the Ge(IV) peak appeared at a potential of −0.796 V. Necessary to obtain a signal is the addition of a complexing agent to the test solution, which was catechol. The optimized method resulted in low detection limits for germanium and gallium of 7.99 × 10^−10^ mol L^−1^ and 3.59 × 10^−10^ mol L^−1^, respectively, with an accumulation time of 60 s [50]. In addition, all three methods were tested for their efficiency in determining these ions in real samples, as discussed in the next section.

## 5. Practical Applications of Gallium(III) Determination

In most of the papers reviewed, the procedures developed for the voltammetric determination of gallium were tested for their analytical application on real samples. Table 2 and Table 3 summarize the data obtained from the practical application of these procedures. Gallium ions were tested in samples of different origins, such as food products, aluminum samples, environmental samples, synthetic samples, and certified reference materials. The analytical usefulness of the developed procedures can be assessed based on the results collected in the tables. Table 2 shows the results obtained from the determination of gallium(III) in food and industrial products. Wheat flour, rice, and wheat were used as food products in which the gallium content was determined [51]. In contrast, reference [43] determined the gallium content of aluminum samples. Gallium determination studies were also carried out using techniques such as AAS and GF−AAS. This was performed to compare the values obtained with these methods. By comparing the results of these determinations, it can be concluded that they confirm the suitability of the developed method for practical application.

Certified material with known concentrations of gallium(III) was used to assess the suitability of the method for the determination of gallium in real samples. Using adsorptive stripping voltammetry with DDSV as a complexing agent, a reference material was used where the manufacturer suggested that gallium was present at a concentration of (1.0 ± 0.4) × 10^−6^ mol L^−1^. By performing a triple measurement according to the developed procedure, the authors were able to determine gallium at a concentration of (9.9 ± 0.5)×10^−7^ mol L^−1^, confirming the usefulness of the developed procedure [47]. In [52], certified reference materials containing no gallium(III) were used to verify the procedure described. The SPS−WW1 wastewater, containing 13 elements, and SPS−SW1 surface water, containing up to 45 elements of known concentration, were used. The use of these CRMs is very important because of their matrix, which is similar to environmental samples. Measurements were repeated three times using the standard addition method, and the recoveries obtained ranged from 92.8% to 98.8%, with a relative standard deviation of 5.6% to 6.2%. These results demonstrate that the method can be applied to the determination of gallium in environmental samples with a complex matrix [52].

The anodic stripping voltammetry method has been tested for the determination of gallium in synthetic samples. The synthetic samples chosen were 2% U−Ga and 10% U−Ga, where the gallium content in these samples was 20 mg g^−1^ and 100 mg g^−1^, respectively. A sample containing 10% gallium could be measured without prior preparation, whereas a sample containing 2% gallium required solvent extraction due to the high interference from uranium. The average gallium content obtained from the five measurements was 19.822 mg g^−1^ for the 2% U−Ga sample and 99.114 mg g^−1^ for the 10% U−Ga sample. This confirms that the developed method has very good precision and high accuracy for the determination of gallium [42].

Table 3 also shows the results obtained in the determination of Ga(III) in samples such as tap water, river water, and certified reference materials. As confirmed in reference [45], in which a solid bismuth microelectrode was used as the working electrode, the suitability of the voltammetric method for the determination of gallium is confirmed by the recoveries obtained, which range from 92.4% to 105.5%. It should also be noted that the actual samples of both surface water and CRMs were not specially prepared for the measurements, which means that the duration of the tests performed is not extended [45]. Piech, in his procedure for the determination of gallium using the Hg(Ag)FE electrode, described the application of this method to the analysis of real samples, such as tap water, water taken from the Rudawa and Vistula rivers, and sediment. Table 3 shows the concentrations of gallium added and determined by this method in these samples. In the case of water samples from the Rudawa and Vistula Rivers and sediment, measurements taken without the addition of a known concentration of gallium showed the presence of this ion in these samples. This indicates that the gallium concentration at these sites is at the level of quantification for the method in question. Subsequent additions of known concentrations of gallium(III) increased the determined concentration in proportion to this addition, demonstrating the good accuracy and linearity of the method. The results obtained confirmed that the method can be successfully applied to the determination of gallium in environmental samples, offering high sensitivity and the possibility to monitor contaminants in water and sediments. In addition, the ability to easily regenerate the Hg(Ag)FE electrode makes this method practical and efficient for field analysis, making it an attractive alternative to other analytical techniques [49].

One paper suggested introducing resin into the environmental samples being analyzed to remove organic matter that can interfere with the signal. Water samples were mixed with Amberlite XAD−7 resin to remove organic matter that could interfere with the signal. In the solutions tested, the presence of the labeled ions was not detected in concentrations detectable by this method, so to confirm its accuracy, the samples analyzed were fortified with these ions, and recovery studies were carried out. The recoveries for gallium ranged from 95.3 to 98.7% and for indium from 94.3 to 97.6%, confirming the feasibility of using this method for the determination of real samples [53]. In a second method described in [41] for the simultaneous determination of gallium and indium, the presence of these ions was investigated in an InGa alloy of known composition. In the alloy sample analyzed, 66.2% In and 33.5% Ga were detected, while the actual content was 66.7% In and 33.3% Ga. The results obtained demonstrate the excellent precision of the method developed by Meavecky et al. [41]. Another study tested the effectiveness of the method in analyzing various environmental samples such as river water, tap water, and soil. The use of the Hg(Ag)FE electrode allowed recoveries ranging from 97% to 106% for gallium and from 96% to 112% for germanium. These results confirm the high accuracy and suitability of this method for the analysis of real samples [50].

## 6. Conclusions

With the development of industrial and technological applications of gallium, the likelihood of environmental contamination increases, requiring sensitive and reliable analytical methods for its detection. This review of voltammetric methods for the quantitative determination of gallium(III) provides a concise and comprehensive summary of their analytical characteristics and applicability. Among electrochemical techniques, stripping voltammetry is characterized by high sensitivity, low detection limits, and suitability for the analysis of complex matrices.

Various techniques have been described in the literature for the determination of gallium at low concentrations. One of the most widely used methods is inductively coupled plasma mass spectrometry (ICP-MS), which is considered the gold standard for trace metal analysis due to its exceptional sensitivity, selectivity, and wide range of quantification. ICP-MS allows the detection of gallium at the ultra-trace level, reaching detection limits of 10^−13^ mol L^−^¹. However, high operating costs, complex sample preparation, and the need for sophisticated laboratory equipment make the method less accessible for everyday environmental monitoring or field analysis. For this reason, stripping voltammetry (SV) is a promising alternative as it is cheaper, more portable, and easier to use. Although the detection limits of this technique are slightly higher than those of ICP−MS, the advantages of SV are making it increasingly popular, especially in the context of field analysis and environmental monitoring.

Different types of working electrodes are presented, including mercury-based and mercury-free alternatives, each with unique advantages and limitations. The continued development of new materials used in the construction of working electrodes contributes to the advancement of gallium determination techniques. The working electrodes have been further modified by coating them with films that improve the gallium accumulation capacity, thereby increasing the sensitivity and selectivity of the analysis. The lowest detection limit for gallium was obtained with the HMDE electrode, while the second lowest detection limit was obtained with the MWCNT/SGCE electrode, which was additionally modified with a lead film. Regarding sensor reuse, electroanalytical sensors can be used repeatedly for analysis, provided they are properly maintained. Their durability and effectiveness depend on proper cleaning and protection from mechanical damage. In practice, they can be used for long periods, making them a cost-effective solution for routine analysis.

In AdSV methods, it is necessary to introduce a suitable complexing agent to ensure the accuracy of the measurements. In the case of gallium determination, cupferron was found to be the most effective complexing agent, and it was used with a variety of working electrodes. The use of a suitable complexing agent also effectively eliminates interference from other ions, which is crucial for accurate measurements. Such behavior was reported in [43], where DDTC, as a complexing agent, masked the negative effects of zinc and copper on the gallium signal.

The influence of factors such as the presence of interfering ions and organic matter on gallium determination is also discussed. There is a wide range of substances in natural samples that can significantly affect the gallium signal, making accurate analysis challenging. Conclusions from the analysis of the available data indicate that these interferences can be minimized by the use of optimized analytical techniques and appropriate masking agents. For example, the presence of Fe(III) and Al(III) ions can cause a significant decrease in the gallium signal, but their effect can be effectively reduced by the addition of F^−^ ions to the test solution. This indicates the important role of such modifications in improving the accuracy of measurements in the presence of interfering metallic ions. Furthermore, most of the foreign ions have no effect on the gallium signal, demonstrating the good selectivity of the developed gallium determination methods. Suggested methods to eliminate organic interference included mixing the sample with resin and mineralizing the sample prior to measurement. Effective elimination of interference caused by the presence of the surfactant Triton X−100 was achieved by pre-mixing the sample with Amberlite XAD−16 resin. It was shown that interference caused by Triton X−100 did not occur when its concentration in the sample was 20 mg L^−1^, provided that the sample was pretreated with the resin. In contrast, the complete disappearance of the gallium signal was observed at a Triton X−100 concentration as low as 0.5 mg L^−1^ in samples that did not undergo such preparation [48]. These results underscore the effectiveness of a sample resin premixing procedure in eliminating interferences. Mixing the sample with resin is a better option due to the shorter preparation time and less complexity of the process as it is not complicated or time-consuming.

This review also covers the practical applications of the procedures discussed, demonstrating their effectiveness in the analysis of real samples, such as water quality monitoring and the analysis of biological samples. Samples of known compositions containing specific concentrations of gallium were also tested, as well as certified reference materials with a complex matrix containing no gallium ions. In such cases, it was necessary to add known concentrations of gallium to the samples and perform a recovery analysis. Satisfactory results were obtained for all samples analyzed, including food (wheat flour, rice, wheat), tap water, river water (Rudawa, Vistula, Bystrzyca), sediment, and aluminum samples (aluminum cans, foils, and containers). In addition, certified reference materials (SPS−SW2 surface water, SPS−WW1 wastewater) and synthetic gallium(III) solutions were analyzed. The results obtained confirmed the high efficiency of the methods used for the determination of gallium in environmental samples as well as in complex industrial and biological matrices.

In conclusion, further development and optimization of voltammetric methods and other electroanalytical techniques are key to ensuring even greater precision and efficiency in the determination of gallium in a wide range of applications, from environmental monitoring to industry and biotechnology. Future research should focus on improving these methods to enable their application to even more diverse samples and areas of analytical chemistry. An important direction of development is the development of methods that minimize interferences and lower detection limits. The introduction of new electrode materials will make the determination of gallium easier, more efficient, and more laboratory-friendly.

## Figures and Tables

**Table 1:** Summary of voltammetric stripping techniques for the determination of gallium. The methods were ranked in ascending order based on their limit of detection.

**Method**	**Working Electrode**	**Supporting Electrolyte**	**Complexing Agent**	**LoC** **[mol L^−1^]**	**LoD** **[mol L^−1^]**	**Accumulation Time** **[s]**	**Investigated Interferents**	**Application**	**Ref.**
ASV	HMDE	0.02 mol L^−1^ NaClO_4_, 0.005 mol L^−1^ CH_3_COOH, рН 3.2	-	nd	5.7 × 10^−11^	300	Cd(II), Pb(II), Zn(II), Cu(II)	-	[40]
AdSV	PbFE/MWCNT/SGCE	0.1 mol L^−1^ acetate buffer, pH 5.6	cupferron	3.0 × 10^−9^–4.0 × 10^−7^	9.5 × 10^−10^	60	Al(III), Bi(III), Ca(II), Cd(II), Cr(III), Cr(VI), Co(II), Cu(II), Fe(III), Mg(II), Mn(II), Ni(II), Zn(II), Mo(VI), Sb(III), Ti(IV), V(V)	tap water, Bystrzyca River, CRM	[54]
AdSV	Hg(Ag)FE	0.1 mol L^−1^ acetate buffer, pH 4.8	catechol	1.25 × 10^−9^–9.0 × 10^−8^	3.6 × 10^−10^	60	Mn(II), Pb(II), Cd(II), Bi(III), Sb(V), As(III), Zn(II), Cu(II), Fe(III) Triton X−100, HA	tap water, Rudawa River, Vistula River, soil	[50]
AdSV	CPE	0.1 mol L^−1^ NH_4_OAc−HCl buffer, pH 4.5	ARS	2.9 × 10^−10^–8.6 × 10^−8^	1.4 × 10^−10^	180	Ni(II), Mg(II), Ca(II), Cd(II), Zn(II), Mn(II), Ba(II), Pb(II), Co(II), Mo(VI), As(III), V(V), Se(IV), Cr(III), Bi(III), Zr(IV), Sn(IV), Fe(III), Al(III), La(III), Sc(III), Y(III)	wheat flour, rice, wheat	[51]
AdSV	HMDE	0.1 mol L^−1^ acetate buffer, pH 3.0	cupferron	5.0 × 10^−10^–5.0 × 10^−7^	1.3 × 10^−10^	30	As(III), Ca(II), Cd(II), Co(II), Mn(II), Ni(II), Zn(II), Tl(I), Al(III), As(V), Cr(III), Cr(VI), Cu(II), Ge(IV), Mg(II), Pb(II), Se(VI), W(VI), Bi(III), Fe(III), Hg(II), Sb(III), Mo(VI), Se(IV) CTAB, SDS, Triton X−100, HA, FA	Lake Zemborzyce, Bystrzyca River	[48]
AdSV	Hg(Ag)FE	0.1 mol L^−1^ acetate buffer, pH 4.8	catechol	2.0 × 10^−9^–1.0 × 10^−7^	1.0 × 10^−10^	90	s Mn(II), Pb(II), Cd(II), Bi(III), Sb(V), As(III), V(V), Zn(II), Cu(II), Fe(III), Ge(IV) Triton X−100, HA	tap water, Rudawa River, Vistula River, soil	[49]
AdSV	BiFE/GCE	0.1 mol L^−1^ acetate buffer, pH 5.0	cupferron	2.5 × 10^−8^–1.5 × 10^−6^	9.7 × 10^−9^	30	-	Bystrzyca River, Lake Zemborzyce	[53]
ASV	BiFµE	0.1 mol L^−1^ acetate buffer, pH 4.6	-	2.0 × 10^−8^–2 × 10^−6^	7.0 × 10^−9^	60	Al(III), Cu(II), Hg(II), Sn(II), Pb(II), V(V), Ti(IV), Cd(II), Mo(VI), Fe(II), Zn(II) SDS, CTAB, Triton X−100, HA, FA, NOM, rhamnolipid	CRMs, tap water, Bystrzyca River	[45]
AdSV	PbFE/GCE	0.05 mol L^−1^ CH_3_COOH	cupferron	1.0 × 10^−8^–2.0 × 10^−7^	3.8 × 10^−9^	60	Al(III), Bi(III), Ca(II), Cd(II), Cr(III), Cr(VI), Co(II), Cu(II), Fe(III), Mg(II), Mn(II), Ni(II), Zn(II)	CRMs	[52]
ASV	Hg(Ag)FE	0.01 mol L^−1^ KSCN, pH 3.05	-	5.0 × 10^−9^–8.0 × 10^−8^	1.4 × 10^−9^	120	Pb(II), Cd(II), Tl(I), Mn(II), Se(IV), In(III), Bi(III), Ge(IV), Th(IV), U(VI), Mo(VI), Zn(II), Cu(II), Fe(III),	aluminum samples	[43]
AdSV	SMDE	0.2 mol L^−1^ acetate buffer, pH 4.8	SVRS	0–2.3 × 10^−7^	1.1 × 10^−9^	60	Bi(III), Al(III), Cu(II), Sn(IV), Pb(II), Mn(II), Ni(II), Ca(II), Ba(II), Mg(II), Fe(III), Cd(II), Hg(II), In(III), Ti(IV), Zn(II), Ni(II) gelatin	-	[46]
ASV	BiFE/GCE	0.2 mol L^−1^ acetate buffer, pH 4.6	-	2.9 × 10^−7^–1.4 × 10^−6^	9.5 × 10^−8^	240	Cd(II), Cu(II), Zn(II), Tl(I)	water spiked with Ga(III)	[44]
AdSV	HMDE	(1) acetic−acetate buffer, pH 4.0 (2) Britton–Robinson buffer, pH 3.0 (3) acetic−acetate buffer, pH 5.5	(1) APDC (2) PCV (3) DDTC	(1) 5.0 × 10^−8^–2.7 × 10^−7^ (2) 5.0 × 10^−9^–4.8 × 10^−7^ (3) 1.0 × 10^−9^–2.1 × 10^−7^	(1) 5.0 × 10^−8^ (2) 9.9 × 10^−9^ (3) 1.3 × 10^−9^	(1) 50 (2) 100 (3) 30	Ag(I), Ca(II), Mg(II), Sb(V), Sr(II), Cu(II), As(V), Mn(II), Cd(II), Al(III), Bi(III), Pb(II), Tl(I), In(III), Fe(III), Ni(II), Zr(IV), Ba(II), Sb(III), As(III), Cr(III), Cr(VI), Co(II), Zn(II)	certificate water sample, tap water sample spiked with Ga	[47]
ASV	SMDE	0.5 mol L^−1^ NaSCN + 4.2 mol L^−1^ NaClO_4_, pH 2.0	-	3.0 × 10^−7^–1.0× 10^−6^	1.0 × 10^−8^	360	-	-	[39]
ASV	MFE/GCE	0.5 mol L^−1^ NaSCN + 1 mol L^−1^ NaClO_4_, pH 2.0	-	1.0 × 10^−7^–1.0× 10^−6^	-	100	-	Synthetic U−Ga samples	[42]

HMDE—hanging drop mercury electrode; MWCNT/SGCE—multiwall carbon nanotubes/spherical glassy carbon electrode; Hg(Ag)FE—renewable mercury film silver-based electrode; CPE—carbon paste electrode; BiFE—bismuth film electrode; BiFµE—solid bismuth microelectrode; PbFE—lead film electrode; SMDE—static mercury drop electrode; MFE—mercury film electrode; ARS—alizarin red S; SVRS—solochrome violet RS; APDC—ammonium pyrrolidine dithiocarbamate; PCV—pyrocatechol violet; DDTC—diethyldithiocarbamate2. Role of working Electrodes for Ga(III) determination.

**Table 2:** Accuracy evaluation of the voltammetric method for the determination of Ga(III) in some food and industrial samples with reference to the AAS−GFAAAS method.

**Sample**	**Found ASV** **[µg g^−1^]**	**Found GF−AAS** **[µg g^−1^]**	**Ref.**
Aluminum can	8.94	10.1	[43]
Aluminum foil	24.3	24.9	
Aluminum foil container	33.1	30.2	
**Sample**	**Found AdSV** **[µg g^−1^]**	**Found AAS** **[µg g^−1^]**	**Ref.**
Wheat flour	0.118	0.123	[51]
Rice	0.148	0.142	
Wheat	0.127	0.122	

**Table 3:** Determination of Ga(III) in different samples by stripping voltammetry method.

Method	Sample	Added (nmol L^−1^)	Found (nmol L^−1^)	Ref.
AdSV	Tap water	0.0	0.0	[49]
		1.5	1.55	
		5.0	11.1	
	Rudawa River	0.0	1.19	
		1.5	2.58	
		5.0	6.25	
	Vistula River	0.0	1.02	
		1.5	2.602	
		5.0	5.96	
	Sediment	0.0 ppm	20.9 ppm	
		20 ppm	40.1 ppm	
AdSV	Tap water	0.0	0.0	[54]
		45.0	46.8	
		65.0	68.2	
		110.0	107.8	
	Bystrzyca River water	0.0	0.0	
		45.0	42.9	
		65.0	63.0	
		110.0	114.9	
	Certified reference material SPS−SW2 surface water	0.0	0.0	
		45.0	43.3	
		65.0	66.7	
		110.0	113.5	
ASV	Certified reference material SPS−WW1 wastewater	100.0	92.4	[45]
		400.0	382.4	
	Certified reference material SPS−SW2 surface water	50.0	52.0	
		200.0	211.0	
	Bystrzyca River water	100.0	94.4	
		400.0	384.8	
	Tap water	50.0	51.5	
		200.0	195.0	
ASV	Samples spiked by of Ga(III)	5.0	4.7	[43]
		12.5	12.1	
		18.0	18.2	
ASV	Water	359	374	[44]
		359	377	
		287	287.23	
ASV	Synthetic sample of gallium(III) solutions	618	612	[42]
		1160	1150	
		2250	2230	
		3690	3660	

## Data Availability

Not applicable.
